# Strengths and Weaknesses of the Gray Mouse Lemur (*Microcebus murinus*) as a Model for the Behavioral and Psychological Symptoms and Neuropsychiatric Symptoms of Dementia

**DOI:** 10.3389/fphar.2019.01291

**Published:** 2019-10-30

**Authors:** Fabien Pifferi, Jacques Epelbaum, Fabienne Aujard

**Affiliations:** ^1^UMR CNRS/MNHN 7179, Mécanismes Adaptatifs et Evolution, Brunoy, France; ^2^Unité Mixte de Recherche en Santé 894 INSERM, Centre de Psychiatrie et Neurosciences, Université Paris Descartes, Sorbonne Paris Cité, Paris, France

**Keywords:** *Microcebus*, primate model, aging, Alzheimer’s disease, cognition, circadian rhythms

## Abstract

To face the load of the prevalence of Alzheimer’s disease in the aging population, there is an urgent need to develop more translatable animal models with similarities to humans in both the symptomatology and physiopathology of dementia. Due to their close evolutionary similarity to humans, non-human primates (NHPs) are of primary interest. Of the NHPs, to date, the gray mouse lemur (*Microcebus murinus*) has shown promising evidence of its translatability to humans. The present review reports the known advantages and limitations of using this species at all levels of investigation in the context of neuropsychiatric conditions. In this easily bred Malagasy primate with a relatively short life span (approximately 12 years), age-related cognitive decline, amyloid angiopathy, and risk factors (i.e., glucoregulatory imbalance) are congruent with those observed in humans. More specifically, analogous behavioral and psychological symptoms and neuropsychiatric symptoms of dementia (BPSD/NPS) to those in humans can be found in the aging mouse lemur. Aged mouse lemurs show typical age-related alterations of locomotor activity daily rhythms such as decreased rhythm amplitude, increased fragmentation, and increased activity during the resting-sleeping phase of the day and desynchronization with the light-dark cycle. In addition, sleep deprivation successfully induces cognitive deficits in adult mouse lemurs, and the effectiveness of approved cognitive enhancers such as acetylcholinesterase inhibitors or N-methyl-D-aspartate antagonists is demonstrated in sleep–deprived animals. This result supports the translational potential of this animal model, especially for unraveling the mechanisms underlying dementia and for developing novel therapeutics to prevent age-associated cognitive decline. In conclusion, actual knowledge of BPSD/NPS-like symptoms of age-related cognitive deficits in the gray mouse lemur and the recent demonstration of the similarity of these symptoms with those seen in humans offer promising new ways of investigating both the prevention and treatment of pathological aging.

## Introduction

Common laboratory model organisms—yeast, nematodes (*Caenorhabditis elegans*), fruit flies (*Drosophila melanogaster*), and mice—have helped scientists substantially advance our understanding of neuropsychiatric diseases (Götz and Ittner, 2008; Nestler and Hyman, 2010). However, the limits of such models are particularly pronounced in this field of research. Many of the human symptoms leading to psychiatric diagnoses (e.g., hallucinations, delusions, sadness, guilt) cannot be convincingly reproduced in the common animal models cited above. When reasonable correlates do exist, such as in rodents (e.g., abnormal social behavior, motivation, working memory, emotion, and executive function), the correspondence still remains limited. Moreover, determining how symptoms in a rodent correspond to a recognized human neuropsychiatric disorder is not trivial. According to the *Diagnostic and Statistical Manual of Mental Disorders, 5th Edition* (American Psychiatric Association, 2013), “most diagnoses are based on phenomenology, i.e., on symptoms, signs, and the course of the illness” rather than validated tests. Thus, it is of primary importance to take advantage of the extreme similarity between non-human primates (NHPs) and humans. Indeed, the anatomical and functional organization of NHP brains is homologous to the human brain (i.e., the existence of specialized motor, perceptual, and cognitive abilities not found in rodents) (Borra and Luppino, 2019). Therefore, neuropsychiatric disorders can be better replicated in NHPs than in rodents. However, the use of NHP species, as essential as it appears to be for neuropsychiatric research, has also been a caveat. Increased ethical pressure to regulate the use of animals for scientific purposes is especially strong in the case of primates. In addition to ethical issues, the high cost of breeding, the relatively long life spans, the large body size, and social system constraints are limiting factors that need to be taken into account. Therefore, the use of a smaller-sized NHP species such as mouse lemurs (*Microcebus murinus*) to model age-associated cognitive disorders and neuropsychiatric conditions, as reviewed herein, would be a good compromise. The gray mouse lemur belongs to the Strepsirhini suborder and to the Cheirogaleidae family, composed of small, omnivorous primates. As primates, they have the closest phylogenetic distance to humans, much closer than that of rodents (about mid-distance between mouse and human) (Ezran et al., 2017). These primates are nocturnal, arboreal solitary foragers that sleep in groups during the daytime. In the wild, during the 6 months of the hot rainy season, which is characterized by a long photoperiod, elevated temperatures, and abundant food resources, the mouse lemur exhibits a high level of activity and has a high metabolic rate during the dark phase. It corresponds to the mating season. Conversely, the 6 months of the cooler dry season are characterized by harsh conditions in terms of food resources and temperatures. At the onset of the dry season (photoperiod shorter than 12 h), the mouse lemur metabolism slows down, leading to an increase in fat deposits and the occurrence of pronounced daily phases of hypometabolism (Schmid and Speakman, 2000; Génin and Perret, 2003). These physiological changes are highly dependent on the photoperiod (Perret and Aujard, 2001; Génin and Perret, 2003). In captivity, gray mouse lemurs can live up to 13 years (Pifferi et al., 2019), while their lifespan in the wild is significantly shorter (Lutermann et al., 2006). In this study by Lutermann et al., no animals older than 6 years old were observed. In addition, due to their relatively small body size (head-body length of approximately 15 cm) and low body mass (60–80 g), mouse lemurs can be easily bred and kept in captivity at low costs. This species, thus, would be a good compromise between practical breeding methods and physiological and phylogenetic proximity to humans. In addition, this species offers a natural biological heterogeneity and spontaneous occurrence of pathologies, especially age-related neurodegenerative diseases. Thus, even though some limitations need to be considered, this species is considered an emerging model organism for biology, behavior, and health studies (Ezran et al., 2017; Roberts, 2019), particularly in the context of aging (Bons et al., 2006; Austad and Fischer, 2011; Finch and Austad, 2012; Laurijssens et al., 2013).

## Potential Markers of Behavioral and Psychological Symptoms of Dementia in Gray Mouse Lemurs of All Ages

### Cognition and Sensory–Motor Functions

The mouse lemur is a primate that has been extensively studied in relation to cognitive function and its evolution during aging (Languille et al., 2012b). Cognitive functions have been mainly studied in young and adult, male mouse lemurs and studies covered the following major cognitive domains: recognition, spatial or working memories, stimulus reward associative learning, and set-shifting performances. Such cognitive tasks rely on brain function and sensory–motor functions. Age-related effects on the sensory functions of mouse lemurs have been studied in this context and as markers of aging.

The overall pattern of cognitive performance throughout aging reflects changes similar, in many aspects, to those that occur in human aging. This similarity is not found in most rodents, whose memory function does not match that of humans (Deacon, 2014). One specific rodent species, *Octodon degus*, has more similarities with humans and can provide benefits in aging studies (Tarragon et al., 2013; Hurley et al., 2018). In most tasks, the acquisition of the rule is not impacted by aging (Picq, 1998; Picq and Dhenain, 1998), and cognitive processes involved in simple stimulus-reward association performances are preserved during aging (Joly et al., 2006; Picq, 2007). In tasks influenced by anxiety, aged mouse lemurs actually performed better than young ones (Picq, 1993; Némoz-Bertholet and Aujard, 2003; Languille et al., 2015) (see Anxiety Section for details). In contrast, retention capacity or new object memory expresses deficits with aging (Picq, 1998; Picq and Dhenain, 1998; Picq et al., 2015). In fact, the ability to form simple stimulus-reward associations is mainly preserved with aging, while working memory and the ability to shift strategies are impaired in most aged mouse lemurs. Interestingly, in some tasks such as those testing reference spatial memory, spatial and object recognition memory, and the capacity to use flexibly acquired information (generalization and spatial rule-guided discrimination tasks), impairment is only observed in a subset of aged mouse lemurs. In some cases, even very old animals perform as well as young ones, while some middle-aged animals show drastic cognitive deficits. This high interindividual variability mimics, to some extent, the pattern of cognitive aging described in humans (Salthouse, 2017; Barter and Foster, 2018). It allowed to clearly distinguish good from bad performers in cognitive tasks (Languille et al., 2015). This variability is a fruitful background for exploring discriminant cognitive markers of behavioral and psychological symptoms of dementia (BPSD), and it also brings possibilities to search for physiological and neural correlates of normal vs pathological aging. Due to the relatively long life span of mouse lemurs compared to rodents, interindividual variability during middle age allows the implementation of various protocols for studying the aging process and risk factors for neurodegenerative pathologies. Recent attempts have been made to link cognitive function with fitness in wild mouse lemurs, showing that cognitive performance can be used to explain evolutionary trajectories and that the ecology of a species is highly relevant for understanding the consequences of individual differences in cognitive abilities (Huebner et al., 2018). Some personality traits have been linked to some life history traits, such as body weight at birth, showing that some behavioral variations can account for gene dispersal models and, thus, selection (Thomas et al., 2016). Finally, recent evidence of a correlation between glucose homeostasis impairment and cognitive deficits in middle-aged mouse lemurs reinforces the potential role of metabolic function as a risk factor for pathological aging (Djelti et al., 2016), as is the case in humans (Geijselaers et al., 2015). This link represents an interesting opportunity for research on type-2 diabetes as a risk factor for neurodegenerative diseases, since mouse lemurs express age-related glucose metabolism disorder, among other systemic disorders, that resemble those observed in humans. Similar to humans, impaired fasting blood glucose in the mouse lemur was associated with cognitive impairment and cerebral atrophy in middle-aged animals (Djelti et al., 2016). This relationship is confirmed by the positive impact of caloric restriction or micronutrient supplementation on both cognition (at least during the first years of treatment) and glucoregulatory functions. In the mouse lemur, cognitive performance is enhanced during chronic caloric restriction or resveratrol supplementation, which is linked to lower glucose intolerance and insulin response (Dal-Pan et al., 2011).

In 2007, the incidence of cataracts in mouse lemurs was published (Beltran et al., 2007). This research revealed a relatively high incidence of bilateral, progressive cataracts in aged mouse lemurs, with some starting as early as middle age. This incidence rate is not far from that observed in humans (Klein and Klein, 2013). Since then, all animals involved in cognitive tasks requiring vision have been regularly checked for vision. Motor performance and equilibrium are of major interest for their impact on cognitive task performance and in the aging process. In mouse lemurs, physical activity and jumping reduced and motor performance on a rotarod is worse in aged animals (Némoz-Bertholet and Aujard, 2003; Némoz-Bertholet et al., 2004). Olfaction, which plays a major role in communication, reproductive physiology, and mating in this nocturnal species, shows a significant decline with age (Némoz-Bertholet et al., 2004; Cayetanot et al., 2005). This finding deserves more investigation with regard to aging, since olfaction is one of the earliest sensory deficits preceding the emergence of Alzheimer’s disease (AD) (Murphy, 2019).

### Anxiety

In humans, anxiety disorders have an early onset [half of all lifetime cases start by age 14 and three-fourths have begun by age 24, according to Kessler and colleagues (Kessler et al., 2005)]. Anxiety is a component of a variety of diseases and appears to be associated with accelerated aging (Perna et al., 2016). No conclusion about causality between anxiety and accelerated aging can be drawn, and in this context, animal models could be useful for deciphering the potential mechanisms of this association. Several studies have focused on anxiety-linked behavior in gray mouse lemurs using mainly rodent-like apparatuses adapted to the specificities of the species. In a 2010 study, Trouche and colleagues (Trouche et al., 2010) used a sequential choice task based on a three-panel runway and tested the age-related differences in a procedural memory task with or without visual cues. They observed significant anxiety-related behavioral differences between young and aged animals. Young adult lemurs showed more perseverative errors than aged animals, particularly in the presence of visual cues. According to the authors, the behavioral response of young adult lemurs was influenced by novelty-related anxiety that contributed to perseverative errors during their performance of the task. Conversely, aged lemurs showed fewer perseverative errors and rapid habituation to the three-panel runway maze but made more memory errors. Overall, these findings are in accordance with observations made in other anxiety-related situations, such as the open field test. In a study by Languille and colleagues (Languille et al., 2015), the latency to the first movement in the open field, a parameter recognized as a marker of anxiety (Dal-Pan et al., 2011; Royo et al., 2018), differed significantly with age. In this task (the open field test adapted for mouse lemurs, consisting of an empty squared box of 1 × 1 m), old animals started exploring earlier than middle-aged and young animals, confirming a decrease in novelty-related anxiety in old animals compared to young animals. Similar observations were made in the light-dark plus maze in the same study (Languille et al., 2015). Interestingly, in both studies, an effect of age on the individual variability in anxiety-related parameters is observed. In the three-panel runway task (Trouche et al., 2010), young animals exhibit higher interindividual variation than older animals, as also reported in the Languille study (Languille et al., 2015). This result could be due to i) a very systematic decrease in anxiety-related behavior in older animals (in the Languille and colleagues study, all tested aged animals systematically moved before 800 s had elapsed, while approximately 70% of young animals did not move before 1,800 s had elapsed, which is the duration limit of the test); or ii) a selection bias of old animals: it cannot be excluded that at old age, the surviving animals are those with lower levels of anxiety behavior, which could explain the difference between young and aged animals. In a more recent study (Zablocki-Thomas et al., 2018) assessing the relationship between early life inputs such as low birth weight and personality traits at older ages, Zablocki‐Thomas and colleagues confirmed the difference in anxiety behavior between young and aged mouse lemurs. They performed emergence tests during which they measured the latency for the animals to escape from a small wooden box and return to their home cage. The authors found an effect of birth weight on emergence latency: animals born with a lower birth weight had the fastest emergence. This result is consistent with previous observation in the open field test, in which mouse lemurs with lower birth weight also started exploring earlier (Thomas et al., 2016). It was thus proposed that low body weight newborns were exploring their environment earlier to avoid competition. This hypothesis is supported by the fact that individuals with a shorter emergence latency tend to have higher growth rates. In addition, the authors found that age had an impact on emergence latency, suggesting that adult personality can potentially change during life span. This idea is consistent with field experiments, using open field and novel object tests on wild mouse lemurs, demonstrating that personality was influenced by age (at least in males). In this study, the authors observed that older individuals were bolder and took more risks than younger ones (Dammhahn, 2012). The authors thus proposed that younger males were less prone to take risks because they had not yet reproduced. This theory also applies in laboratory conditions where competition for females is also high. Indeed, generally, only one dominant male (compared to groups of three males) usually engenders all the offspring (Andrès et al., 2003). These compelling data show that anxiety levels can impact a wide range of physiological and behavioral parameters in the mouse lemur. Interestingly, the existence of an age difference in the expression of anxiety between young and aged mouse lemurs is similar to the difference found in humans. Indeed, even if the prevalence of anxiety disorders over the lifespan is debated (Baxter et al., 2013; Miloyan et al., 2014), it seems clear that a difference in the expression of anxiety behaviors exists in aging humans (Wuthrich et al., 2015). Interestingly, a study focusing on age differences in mental disorders in ten different European countries (McDowell et al., 2014) reported a lower prevalence of mood and anxiety disorders in older adults than in young adults in western European countries. Such observations suggest that the mouse lemur is a promising model for studying anxiety disorders across the lifespan.

### Chronobiological Markers of BPSD

Circadian rhythms refer to biological processes that display an endogenous oscillation of about 24 h. The impairment of the time-keeping system is believed to be at the origin of the age-related changes observed in biological rhythms (Bonaconsa et al., 2013). These alterations can directly involve the central circadian clock or associated physiological and behavioral processes, such as activity–rest or temperature rhythms. Studying the effects of age on circadian parameters is particularly pertinent in the gray mouse lemur, which exhibits a much longer life span than most common laboratory rodents and is less subject to social bias than humans and other social primates (Lavery, 2000; Austad and Fischer, 2011; Languille et al., 2012b; Hozer et al., 2019). In humans, aging is associated with changes in the amplitude and temporal organization of several daily rhythms (Hood and Amir, 2017). In elderly people, the alternation of sleep–wake rhythms is affected by the appearance of, and increase in, activity periods during the night resting phase and periods of sleep during the diurnal activity phase (Huang et al., 2011; Lieverse et al., 2011). This phenomenon, defined as rhythm fragmentation, is generally accompanied by mood disorders (depressive syndrome) that can be at least partially treated with light therapy (Lieverse et al., 2011). Lieverse and colleagues (Lieverse et al., 2011) demonstrated that such treatment in elderly individuals with depressive symptoms led to the partial restoration of sleep–wake rhythms and led to an amelioration of mood and sleep quality. Biological rhythms thus constitute a core element of the aging process in humans (Hood and Amir, 2017). Similar observations were made in mouse lemurs. This species is known to express highly marked biological rhythms and, thus, constitutes an adequate model to study this phenomenon. Similar to humans, rest-activity rhythms in lemurs become more fragmented with age, with a notable and significant increase in locomotor activity during the resting period leading to lower amplitudes and fragmentation of rhythms (Aujard et al., 2006). Even if similar observations have previously been made in humans (Hofman and Swaab, 2006), they do not explain whether these alterations are due to reduced sensitivity to light or to changes at the central level of the circadian clock. It has been observed a high incidence of ocular pathologies in more than 7 years old mouse lemurs, what suggests a decrease in light responsiveness through the filtering of short wavelengths (Beltran et al., 2007). It has been demonstrated that short wavelengths are efficient in the synchronization of daily rhythms in mouse lemurs (Gomez et al., 2012). In addition, the impact of aging on circadian rhythms in mouse lemur has been associated with immune system alterations. Plasma levels of interferon-γ [IFN-γ, a pro-inflammatory cytokine acting as an activator of glial cells and involved in the pathogenesis of numerous brain diseases (Blasko et al., 2004)] correlate with age-related impairments in circadian rhythms and survival. High levels of IFN-γ have been associated with shorter lifespan and free-running period, i.e., *tau* (*tau* being the period expressed by a biological system in the absence of environmental cues). In mouse lemur, IFN-γ plasma levels also correlate with impairments of locomotor activity and body temperature rhythms that are characteristic of aging (increased level of diurnal locomotor activity, advanced onset, and delayed occurrence of minimal body temperature) (Cayetanot et al., 2009).

In addition to circadian rhythm alterations, aging is also accompanied with several changes in sleep patterns. In humans, they include an augmentation of sleep fragmentation (more wake events during the resting period) leading to decreased total sleep time, sleep efficiency, and slow-wave sleep (Luca et al., 2015). Comparable observations were made in lemurs. At a young age, this species exhibits a fragmented sleep pattern, with numerous periods of active waking during the light resting period (Pifferi et al., 2012), which is more comparable to patterns seen in small mammals (Van Erum et al., 2019) than in humans. At an older age, alterations in sleep–wake rhythms consist in less activity during the active phase and more wake episodes and duration during the resting phase accompanied by a reduction in slow-wave sleep (Hozer et al., 2019). Mouse lemurs also exhibit a phase advance, resulting in an earlier wake time when light turns on (Pifferi et al., 2012; Hozer et al., 2019). This is comparable to observations made in older humans (Duffy et al., 1998). Thus, mouse lemur can be considered as an appropriate model of age-related sleep rhythm disturbances. As an example, circadian rhythms disruptions in humans are often associated to bipolar disorder. Among potential treatments, lithium and light therapy could be useful for addressing circadian dysfunction in this disorder (Moreira and Geoffroy, 2016; Sarrazin et al., 2018), and our knowledge of the behavioral abilities of mouse lemurs could provide an appropriate model to test such interventions.

## Mouse Lemur as a Model of AD

### The Case of Sporadic AD

Since the seminal study by Bons et al. (1991) reporting that a fraction of aged mouse lemurs over 8 years old displayed dramatic atrophy in the neocortex, hippocampus, basal ganglia, hypothalamus, brainstem, and cerebellum that was associated with a conspicuous increase in the size of the cerebral ventricles, the presence of neuritic plaques, and neurofibrillary changes, many studies have tried to assess the relevance of the model for sporadic AD. In this species, age-associated cognitive impairment occurs in 10% of >7-year-old animals (Languille et al., 2012b), a prevalence similar to that observed in >65-year-old humans (Steenland et al., 2015; Niu et al., 2017). Age-related cerebral atrophy predicts cognitive deficits in mouse lemurs (Picq et al., 2012), while cognitive function is related to brain network atrophy in AD and type 2 diabetes patients and in healthy individuals (Buss et al., 2018). In lemurs, however, brain atrophy starts in the frontal cortex, then progresses to the temporal and/or parietal regions and then, finally, to the occipital cortex (Kraska et al., 2011), while in AD, medial temporal structures (i.e. entorhinal cortex, hippocampus, and parahippocampal gyrus) are predominantly involved early, followed by the spreading of the pathology into the lateral temporal, inferior parietal, and orbitofrontal regions (Rasero et al., 2017). Other biomarkers, such as cerebrospinal fluid amyloid β_1–42_ and β_1–40_ or total- and phosphorylated-Tau, have not been measured in mouse lemurs. Nevertheless, similar to humans, low plasma amyloid β_1–40_ levels are associated with the atrophy of several white matter and subcortical brain regions, while high plasma amyloid β_1–40_ levels are negatively correlated with the density of neurons accumulating amyloid β deposits (Roy et al., 2015; Gary et al., 2018). Interestingly, higher plasma amyloid β_1–40_ levels are observed in the winter season when animals display high numbers of torpor bouts, but seasonality has not been taken into account for plasma amyloid β levels in human subjects (see (Lue et al., 2017; Hanon et al., 2018), for instance). However, clinically significant associations between seasonality and cognition and neurobiological correlates have been reported in older human subjects independently of AD pathology (Lim et al., 2018). In terms of sensory deficits, hearing loss and central auditory dysfunction in humans are associated with a high risk of conversion to dementia 5 to 10 years later (Bakhos et al., 2015), but only mild presbycusis is observed in >7-year-old animals (Schopf et al., 2014). Fifty percent of such animals develop (Beltran et al., 2007) cataracts, while particular visual functions may be selectively impaired in subgroups of AD patients (Kusne et al., 2017). Olfactory disorders represent an early characteristic of the human disease (Velayudhan, 2015), and olfactory memory deficits are present in 25% of >7-year-old animals (Joly et al., 2006).

The neuropathological diagnosis of AD depends on the concomitant presence of senile plaques and neurofibrillary tangles (NFTs) (Hyman et al., 2012). The former is ranked according to Thal phases and the CERAD neuritic plaque scoring system which ranks the density of histochemically identified neuritic plaques in the regions of the neocortex and the latter according to Braak stages (from limbic regions to cortex). Approximately 20% of >5-year-old mouse lemurs develop such neurodegenerative signs. In such animals, diffuse amyloid deposits are often observed in the cerebral cortex and amygdala, but mature neuritic plaques are rather scarcely present (Petiet et al., 2012; Bertrand et al., 2013). Concerning NFTs, neocortical areas are frequently decorated with hyperphosphorylated Tau “NFT-like” structures, even in young mouse lemurs, whereas the subiculum and entorhinal cortex are only occasionally involved in > 8-year-old animals (Delacourte et al., 1995). The relative sparing of the hippocampus in mouse lemurs contrasts sharply with the high NFT load invariably present in this structure in human Alzheimer’s brains (Braak and Braak, 1991). The presence of “NFT-like” phosphorylated Tau in young animals may be related to the fact that mouse lemurs are one of the few heterothermic primate species. Indeed, hibernators, such as Arctic ground squirrels and Syrian hamsters, show the reversible formation of highly phosphorylated and dephosphorylated states during hibernation bouts and arousals, respectively (Stieler et al., 2011). Another neuropathological feature frequently observed in Alzheimer’s patient brains and, more generally, in older subjects is amyloid angiopathy. This condition is also recorded in 70% of >5-year-old animals (Bertrand et al., 2013). Unfortunately, data are missing concerning synaptic loss in the cortex of cognitively impaired mouse lemurs, a phenomenon that is frequently observed in patients (Finch and Austad, 2012). The progressive loss of limbic and neocortical cholinergic innervation (Hampel et al., 2018) and the dysfunction of somatostatin-positive interneurons associated with memory deficits have also not been clearly demonstrated (Dournaud et al., 1994). Nevertheless, the acetylcholinesterase inhibitor donepezil—and the N-methyl-D-aspartate antagonist memantine—prevent sleep deprivation (SD)–induced deficits in the retrieval of spatial memory both in young and aged mouse lemurs (Rahman et al., 2017) (see Sleep Deprivation to Induce Transient Cognitive Impairment Section for details). Anti-amyloid β immunotherapy induced an immune response, increased amyloid β_1–40_ plasma levels, and elicited microhemorrhages and iron deposits in the choroid plexus (Joseph-Mathurin et al., 2013). Concerning transcriptomics, a single study pinpointed 47 genes discriminating young animals from healthy old animals and “AD-like” animals, particularly genes involved in protein synthesis pathways (Abdel Rassoul et al., 2010), while amyloid precursor protein (APP) metabolism, tau protein binding, lipid metabolism, insulin-like growth factor 1 signaling, and immune response genes are commonly reported in human patients (George et al., 2017; Wingo et al., 2019).

Finally, AD is a heterogeneous disease depending on environmental and genetic risk factors. Genome-wide association studies have now identified 25 different loci associated with the disease (Kunkle et al., 2019), the most important one being the apolipoprotein E4 allele (Genin et al., 2011). In the *M. murinus* genome, only one ancestral allele, closer to apolipoprotein E4, exists (Calenda et al., 1995), with only one nucleotide differing from the human sequence (Salazar et al., 2016). In addition, phylogenetic analysis of two other proteins involved in AD (presenilin 1 and tau) also exhibited higher homology between mouse lemur sequences and human sequences than to any natural rodent model (Salazar et al., 2016).

### Experimental Transmissibility of AD-Like Pathology

If the expression of an AD-like pathology in the mouse lemur has been demonstrated (Bons et al., 2006), the relatively low number of animals that express the pathology (∼10%) (Bons et al., 1991; Bons et al., 2006) supports the interest in developing a model of pathology induction (Gary et al., 2017). A recent program of the experimental transmission of AD-like pathology has been tested in adult mouse lemurs by Dhenain and colleagues (Gary et al., 2019). In this study, AD patient brain homogenates were microinjected into the brains of adult animals without clinical signs of pathology in the beginning of the study. These mouse lemurs were compared to animals injected with control brain homogenates. One year post-inoculation, animals that received AD brain homogenates exhibited significant cognitive impairments, electroencephalographic activity alterations, progressive cerebral atrophy (spreading far from the injection site), and neuronal loss in both the hippocampus and entorhinal cortex. These animals also displayed more β-amyloid depositions, as well as more hyperphosphorylated Tau “NFT-like” structures, than control-inoculated animals. In contrast to brain atrophy, β-amyloid and “NFT-like lesions were only present in regions close to the initial injection sites and were never detected in animals inoculated with control brain homogenates. This result demonstrates that inoculation with AD brain homogenates systematically induced pathognomonic signs that thoroughly mimicked an AD-like pathology in this primate. This result is of primary importance, since it makes the model available for future research projects on AD and will avoid the long-lasting process of detecting and selecting animals naturally exhibiting such pathology.

## Interventions Mimicking BPSD/Studying BPSD Markers

### Anxiolytic Effects of Omega-3 Fatty Acids

As described above (Anxiety Section), the exploration of anxiety disorders in lemurs has shown interesting results and has suggested the decreased prevalence of anxiety during aging in this species. Interestingly, several intervention studies assessed the anxiolytic impact of nutritional interventions such as polyunsaturated fatty acid (PUFAs) of the omega-3 (ω3) series. The brain cell membranes of vertebrates, including primates, are highly concentrated in long-chain PUFAs of the ω3 and omega-6 (ω6) series. These PUFAs are mainly represented by docosahexaenoic acid [DHA, 22:6 (n-3)] and arachidonic acid [AA, 20:4 (n-6)] (Alessandri et al., 2004). The role of ω3 fatty acids has been extensively investigated through dietary deficiencies using rodents deprived of any source of ω3 fatty acids during the perinatal period. Such chronic deficiency leads to decreased brain DHA content and is accompanied by major consequences at the neurosensory level (learning, memory, anxiety, and vision). These impairments have been related to modifications in the neurotransmission processes (mainly monoaminergic neurotransmitters) (Chalon, 2006). Studies in rodents demonstrated that chronic ω3 PUFA deficiency increased in particular anxiety (Takeuchi et al., 2003; Fedorova and Salem, 2006), and more specifically when animals were in an anxiogenic situation (Fedorova and Salem, 2006). Harauma and Moriguchi (Harauma and Moriguchi, 2011) demonstrated that dietary ω3 PUFA deficiency in mice increases chronic mild stress-induced anxiety. In line with the above mentioned results, restauration of dietary ω3 PUFA levels in rodents previously fed and raised with an ω3 PUFA-deficient diet, led to a reduction of anxiety and restored control-like fatty acid content of most brain regions (Carrié et al., 2000; Takeuchi et al., 2003). In addition, Enslen and colleagues (Enslen et al., 1991) observed that the exploration of a novel environment was reduced in ω3-deficient rats, confirming the impact of dietary ω3 PUFA levels on anxiety in rats. Similar exploratory behavior was improved in mice receiving an ω3 PUFA supplement (Carrié et al., 2000), supporting the potential major role of ω3 PUFAs on anxiety. In humans, the relationship between ω3 PUFAs intake and anxiety disorders is unclear. Although several studies suggested a relationship between low intakes of long-chain ω3 PUFAs and a higher prevalence of anxiety with stronger symptoms (Natacci et al., 2018; Thesing et al., 2018), to our knowledge, only one study tested the anxiolytic effect of ω3 PUFAs in humans (Su et al., 2018), which concluded that ω3 PUFAs can contribute lower anxiety symptoms. Thus, more research is needed in this domain, and adapted NHP models could be of interest. In a series of studies in mouse lemur, we tested the impact of tuna oil supplementation [containing mainly long chain ω3 PUFAs, under the form of eicosapentaenoic acid (EPA) and DHA] on behavioral, cognitive, and locomotor performances. In a first study, we supplemented young adult animals with ω3 PUFAs for 5 months and demonstrated, for the first time in a NHP species, that ω3 PUFA supplementation lowered both spontaneous locomotor activity and anxiety and concomitantly improved cognitive performances (animals being less anxious in novel environments, they performed better in learning and memory tasks) (Vinot et al., 2011). This result was confirmed in a further study in which the supplementation of young adults lasted longer (12 months) (Pifferi et al., 2015). We reported that 12 months of ω3 PUFA supplementation reduced anxiety in the open field task and concomitantly increased the success rate in a learning and memory task (mainly due to higher adherence to the task than control animals). These results were linked to better glucose transport to the brain (Pifferi et al., 2015). In a more recent study, supplementation in young adults lasted 21 months and showed a similar conclusion of reduced anxiety in various cognitive tasks, including the open field task (Royo et al., 2018). Interestingly, in addition to better glucose uptake to the brain, we were able to measure increased neurogenesis in associated cerebral regions (including the amygdala). Such a measurement is impossible to perform in humans, reinforcing a major point of interest for using NHPs to model human mental health disorders. In addition, since anxiety varies throughout aging, including in lemurs, in which it decreases with age (see Anxiety Section), we assessed the impact of 5 months of ω3 PUFA supplementation on behavioral parameters including exploratory activity, emotional status, and spatial memory in old animals (Languille et al., 2012a). Aged ω3 PUFA-supplemented animals exhibited no change in anxiety levels measured in the open field task, in contrast to young animals (Vinot et al., 2011; Pifferi et al., 2015; Royo et al., 2018), confirming the specificity of the anxiety response in aged animals in this species.

### Sleep Deprivation to Induce Transient Cognitive Impairment

The development of novel therapeutics to prevent cognitive decline during mild cognitive impairment and AD is facing difficulties. There is a translational barrier between rodents and clinical results (Deguil et al., 2013; Laurijssens et al., 2013). The use of NHPs is recognized as being of major interest in this context (Austad and Fischer, 2011; Laurijssens et al., 2013). However, although age-related functional impairments (including cognitive decline) have been described in gray mouse lemurs and correlated well with cerebral atrophy, not all animals exhibit such alterations. For example, it has been observed that about half of aged mouse lemurs display a specific alteration in long-term memory retention but not in learning (both assessed during a visual discrimination task) (Picq et al., 2015). Although this finding adequately mimics the natural differences that also exist in the human population, it might be insufficient when a higher number of animals presenting deficits is required. In this context, strategies have been developed to increase the availability of animals presenting cognitive alterations, such as *via* brain inoculation with brain extract from AD patients (see Experimental Transmissibility of AD-Like Pathology). A far less invasive alternative is the use of SD to induce reversible transient cognitive impairment. SD is a recognized method to induce transient cognitive alteration and has been extensively used in rodents [for review, see (Colavito et al., 2013)]. Numerous studies reported that SD efficiently induces transient cognitive deficits comparable to those observed in patients with AD-like dementia. The cognitive challenge offered by SD has several benefits over other strategies. Its effects are temporary, it is easy to administer in a standardized fashion without specific equipment, it avoids the bias of pharmacological intervention for the lowering of cognitive functions (drug-induced deficits), and it is ethically well accepted, since it does not induce pain or long-term distress. SD, as a cognitive challenge, provides an interesting strategy to induce cognitive impairment and is promising in the context of testing cognition-enhancing drugs. SD in mouse lemurs was first tested in young animals (Rahman et al., 2013) in a spatial learning and memory retrieval task [using a circular platform task inspired by the Barnes maze in rodents (Rosenfeld and Ferguson, 2014)]. In this task, a learning session preceded a 24 h testing session (memory). This first set of experiments demonstrated that SD applied before learning did not affect cognitive performance, whereas when it was before memory testing, it increased the number of errors and the latency time before reaching the exit (Rahman et al., 2013). The disruptive effect of SD on spatial memory retrieval thus constitutes an interesting validated challenge in investigating the impact of new drugs during both normal and pathological aging. The experiment was repeated in aged animals with similar conclusions, but the effects on memory retrieval (impairment) were stronger in aged animals (Rahman et al., 2017). In the Rahman study, two symptomatic AD drugs were tested to verify the validity of the model. Both donepezil (an acetylcholinesterase inhibitor) and memantine (an N-methyl-D-aspartate antagonist), when administered 3 h before the memory session (during the last third of the SD period), prevented the deficits induced by SD in memory retrieval in both young and aged animals (Rahman et al., 2017). The effect was dose dependent (donepezil at 0.1 and 1 mg/kg was efficient, while memantine was efficient at 1 mg/kg and not at 0.1 mg/kg). These results suggest that both memantine and donepezil can be effective in sleep deprived mouse lemurs. It further supports the translational potential of mouse lemur but also demonstrates the utility of this model for further testing therapies in the context of AD and other neuropsychiatric diseases.

## Limitations and Future Challenges

Although the utility of the mouse lemur as a model of neuropsychiatric conditions has been clearly demonstrated, such a model also exhibits some limitations. From a biological point of view, gray mouse lemurs exhibit particularities that are rare in primates and almost absent in humans. In addition to being nocturnal (active during the dark period), mouse lemurs are a highly photoperiodic species with strongly marked seasonal phenotypes (Génin and Perret, 2003). Faced with specific environmental constraints (cold, caloric, and water restriction), they can enter in a facultative state of daily hypometabolism (Storey, 2015). These peculiarities distinguish this species from humans and must be taken into account when designing experiments. In addition, if the anatomical and functional organization of NHP brains is more homologous to the human brain than to the rodent brain, *strepsirrhine primates* share fewer common characteristics with humans than haplorhine primates. This difference is exemplified by a description of the organization of the sensory thalamus and visual midbrain. In (Saraf et al., 2019b), the authors observe that the “thalamic nuclei and their overall layout in mouse lemurs resemble those of other strepsirrhine primates, and, in size, likely resemble the nuclei of early primates, which were also small and nocturnal.” The same group also observed that “mouse lemurs are likely to have fewer cortical areas than most or all monkeys [ … ] and their brains are expected to closely resemble those of early primates” (Saraf et al., 2019a). Conversely, a more recent study of the functional microarchitecture of the visual cortex in the mouse lemur demonstrates that orientation preference maps reveal a common design principle of the primate visual cortex. This finding illustrates that mouse lemurs could be considered as an intermediary model species between rodents and higher primates. In addition, in the context of a study in which we assessed the hypothesis that electroencephalography (EEG) markers of motor and locomotor activity in mouse lemur could reflect the typical movement-related desynchronization of alpha rhythms (8–12 Hz) in humans. We observed that mouse lemurs and humans could share basic neurophysiological mechanisms. The EEG markers used in these study could represent an interesting experimental model for translational basic and applied research in neurophysiology (Infarinato et al., 2015)

From a societal point of view, there is increased ethical pressure to regulate the use of animals for scientific purposes, which is especially strong in the case of primates (Bennett and Panicker, 2016; Official journal of the European Communities. Legislation. 2019). In this context, using mouse lemurs could be considered a limitation but also an alternative. Indeed, ethical pressure in primate research is mainly focused on great apes (Bennett and Panicker, 2016; Official journal of the European Communities. Legislation 2019), which share more common traits with humans. From a practical point of view, although raising mouse lemurs is less expensive than raising larger primates (Austad and Fischer, 2011), the current limited number of animals is another major weakness. Only a few mouse lemur facilities exist in the world, and all of these are located in western Europe and the United States. The largest colony in the world, located in Brunoy (France), comprises ∼450 live animals. The other smaller colonies are located in Montpellier (France), Hanover (Germany), and Durham (USA). Some laboratory/facilities also host some adult mouse lemurs for research purposes but do not breed them (CEA Fontenay-Aux-Roses, France and U. Stanford, CA, USA). This very limited number of animals (not more than 1,000 live animals in 2019), with regards to the 1.9 million animals used for research in 2017 only in France, limits access to aged animals (with the median lifespan of the mouse lemur being approximately 5.5 years (Languille et al., 2012b), aged animals represent less than 50% of the live animals in captivity). Among these animals, only a fraction will exhibit the specific neuropsychiatric alterations/biomarkers of interest. Thus, it seems obvious that more facilities and higher budgets would be needed in the future for the proper development of the model as an effective alternate model for BPSD studies. It is noteworthy that some of the abovementioned facilities have developed expertise in nonlethal neuroimaging techniques such as magnetic resonance imaging (Nadkarni et al., 2018; Nadkarni et al., 2019) and positron emission tomography imaging (Pifferi et al., 2015), which allow longitudinal neuroanatomical and neurobiological investigations in this species and would be particularly relevant in the context of aging.

In addition to drastically increasing the number of animals available, another near future challenge in promoting the mouse lemur as a more efficient and appropriate BPSD model would be developing genetic tools in this species. The first genome assembly of the mouse lemur dates back to 2007 (Larsen et al., 2017). However, recent improvement in its genome assembly (Larsen et al., 2017) and the publication of the complete mitochondrial genome sequence (Lecompte et al., 2016) have paved the way for the development of genetic tools and studies and represent a major and mandatory resource for the future of biomedical research with this species.

## Author Contributions

FP, JE, and FA all contributed to the writing of the manuscript.

## Conflict of Interest

The authors declare that the research was conducted in the absence of any commercial or financial relationships that could be construed as a potential conflict of interest.
